# Dataset on corrosion degradation behaviour of mild steel in developed nano-cutting coolant from agro waste

**DOI:** 10.1016/j.dib.2020.106435

**Published:** 2020-10-21

**Authors:** Olufunmilayo O. Joseph, Sunday A. Afolalu, Joshua Okeniyi, Moses E. Emetere

**Affiliations:** a Mechanical Engineering Department, Covenant University, Ota, Nigeria; b Department of Physics, Covenant University, Ota, Nigeria

**Keywords:** Bi-nano additive, Mild steel, Corrosion, Open circuit potential, Nano-fluid

## Abstract

Investigation of the corrosion degradation behaviour of mild steel in an admixture of coconut shell and egg shell (CS-ES) based nano-fluid was presented in this study. Mild steel coupons were immersed in different concentrations (nine concentrations and the control as a reference) of the developed nano-fluid for a period of 24, 48, 72, 96, 120, 144 and 168 h. Corrosion rate was calculated based on ASTM Standard G1-03 standard practice for preparing, cleaning and evaluation of corrosion test specimens. Open circuit potential measurements (OCP) were also carried out. The potential of the steel samples in the nano-fluid with respect to time was investigated. This dataset could be used in evaluating the performance of mild steel in CS-ES based nano-fluid.

## Specifications Table

**Table:** 

Subject area	Materials Science and Engineering, Production Engineering
More specific subject area	Corrosion Science and Engineering.
Type of data	Table, text and graph.
How data was acquired	Chemical composition of the steel was investigated using the JSM-7600F Schottky Field Emission Scanning Electron Microscope coupled with energy dispersion spectrometer. OCP measurements were taken using a Digi-Ivy potentiostat.
Data format	Raw and analysed.
Experimental factors	Nine concentrations of nano-cutting fluid (1090, 2080, 3070, 4060, 5050, 6040, 7030, 8020, 9010) were used. Exposure time of 24, 48, 72, 96, 120, 144 and 168 hours was used.
Experimental features	10 mild steel plate samples of dimensions 20 mm x 20 mm were dry abraded with emery papers of 80, 180, 320 and 600 μm and thereafter immersed in 100ml of nano-fluid of different concentrations. The coconut shell and egg shell ratio used was 90-10, 80-20, 70-30, 60-40, 50-50, 40-60, 30-70, 20-80 and 10-90 respectively. Corrosion rate was calculated based on ASTM Standard G1-03 standard practice for preparing, cleaning and evaluation of corrosion test specimens
Data source location	Covenant University. Ota. Ogun-State. Nigeria.
Data accessibility	Data are available within this article

## Value of the Data

• Data for the mass loss corrosion rates can be used to predict the corrosion rates of immersed samples.
• Data on the open circuit potential (OCP) can be used to predict the resting potential of the system.
• The data acquired for OCP can be used to predict the thermodynamic tendency of the mild steel material to participate in electrochemical corrosion reactions within the nano-cutting fluid.
• OCP data can be valuable in predicting the optimum concentration of coconut shell and egg shell admixture for noble behaviour.

## 1 Data

The research work engaged the use of coconut shell as agro-waste and egg shell as bio-waste. Tables 1 and 2 shows the composition of the coconut shell and egg shell respectively. The admixture according to the proportion shown in Table 3 resulted into a nano-cutting fluid. The data reported in this paper is from immersion test and open circuit potential measurements. Corrosion degradation behaviour was assessed by comparing the behaviour of mild steel in various concentrations of nano-cutting fluid relative to the control (conventional cutting fluid) test. The method can be applied as a part of the evaluation process on the performance of the developed nano-cutting fluid. The elemental composition analysis of the mild steel used is presented in Table 4. Data for the immersion test or mass loss corrosion rates are presented in Table 5. Table 6 shows the OCP measurements. Anodic and cathodic reactions are in equilibrium at OCP. Lower concentrations of coconut shell in the admixture showed more positive potential values whereas higher concentrations showed more noble behaviour.

**Table 1:** Compositional analysis of nano particles from coconut shell.

Elements	Composition (%)
C Si O Mn Fe Al	44.36 0.20 8.86 0.45 45.82 0.31

**Table 2:** Compositional analysis of nano particles from egg shell.

Elements	Composition (%)
C Na Mn O Ca Al	6.86 12.31 0.47 18.4 57.30 1.23

**Table 3:** Nanoparticle admixture rate.

Sample	Nano particles (%)(Coconut shell)	Nano particles (%)(Egg shell)	Nano fluid (g/l) (Water as base)
1	60	40	0.1
2	30	70	0.2
3	90	10	0.25
4	40	60	0.50
5	50	50	0.75
6	80	20	1.00
7	70	30	1.25
8	10	90	1.50
9	20	80	2.00
10	Control

**Table 4:** Compositional analysis of low carbon steel.

Elements	Composition (%)
C Si Mn Cr Mo Ni AL Cu Co V W Fe	0.11 0.24 0.47 0.12 0.02 0.1 0.03 0.14 0.0012 0.003 0.06 Bal

**Table 5:** Immersion test results.

Corrosion rate (mm/year)							
Sample (CS%/ES%)	24hrs	48hrs	72hrs	96hrs	120hrs	144hrs	168hrs
CS-60/ES-40	0.0072	0.00436	0.00057	0.00137	0.00044	0.0005	0.00019
CS-30/ES-70	0.00088	0.00066	0.00003	0.00003	0.00004	0.00008	0.00012
CS-90/ES-10	0.00036	0.0001	0.00003	0.0004	0.00035	0.00013	0.00022
CS-40/ES-60	0.00168	0.00223	0.00017	0.00012	0.00023	0.00013	0.00013
CS-50/ES-50	0.00703	0.00297	0.00105	0.00096	0.00042	0.0004	0.00012
CS-80/ES-20	0.00403	0.00377	0.00134	0.00124	0.0009	0.0005	0.0002
CS-70/ES-30	0.00965	0.00732	0.00108	0.00206	0.00107	0.00074	0.00054
CS-10/ES-90	0.00547	0.00272	0.00005	0.00079	0.00027	0.0004	0.00035
CS-20/ES-80	0.00404	0.00239	0.00002	0.00001	0.00024	0.00001	0.0001
Control	0.00102	0.00066	0.00021	0.00014	0.0002	0.00021	0.0003

**Table 6:** Open circuit potential results.

Time (sec)	V(V)	V(V)	V(V)	V(V)	V(V)	V(V)	V(V)	V(V)	V(V)
Time (sec)	1090	2080	3070	4060	5050	6040	7030	8020	9010
0.00E+00	−0.49	−0.48	−0.53	−0.5	−0.48	−0.47	−0.47	−0.47	−0.47
2.00E-02	−0.49	−0.48	−0.53	−0.5	−0.48	−0.47	−0.47	−0.47	−0.47
4.00E-02	−0.49	−0.48	−0.53	−0.5	−0.48	−0.47	−0.47	−0.47	−0.47
6.00E-02	−0.49	−0.48	−0.53	−0.5	−0.48	−0.47	−0.47	−0.47	−0.47
8.00E-02	−0.49	−0.48	−0.53	−0.5	−0.48	−0.47	−0.47	−0.47	−0.47
1.00E-01	−0.49	−0.48	−0.53	−0.5	−0.48	−0.47	−0.47	−0.47	−0.47
1.20E-01	−0.49	−0.48	−0.53	−0.5	−0.48	−0.47	−0.47	−0.47	−0.47
1.40E-01	−0.49	−0.48	−0.53	−0.5	−0.48	−0.47	−0.47	−0.47	−0.47
1.60E-01	−0.49	−0.48	−0.53	−0.5	−0.48	−0.47	−0.47	−0.47	−0.47
1.80E-01	−0.49	−0.48	−0.53	−0.5	−0.48	−0.47	−0.47	−0.47	−0.47
2.00E-01	−0.49	−0.48	−0.53	−0.5	−0.48	−0.47	−0.47	−0.47	−0.47
2.20E-01	−0.49	−0.48	−0.53	−0.5	−0.48	−0.47	−0.47	−0.47	−0.47
2.40E-01	−0.49	−0.48	−0.53	−0.5	−0.48	−0.47	−0.47	−0.47	−0.47
2.60E-01	−0.49	−0.48	−0.53	−0.5	−0.48	−0.47	−0.47	−0.47	−0.47
2.80E-01	−0.49	−0.48	−0.53	−0.5	−0.48	−0.47	−0.47	−0.47	−0.47
3.00E-01	−0.49	−0.48	−0.53	−0.5	−0.48	−0.47	−0.47	−0.47	−0.47
3.20E-01	−0.49	−0.48	−0.53	−0.5	−0.48	−0.47	−0.47	−0.47	−0.47
3.40E-01	−0.49	−0.48	−0.53	−0.5	−0.48	−0.47	−0.47	−0.47	−0.47
3.60E-01	−0.49	−0.49	−0.53	−0.5	−0.48	−0.47	−0.47	−0.47	−0.47
3.80E-01	−0.49	−0.49	−0.53	−0.5	−0.48	−0.47	−0.47	−0.47	−0.47
4.00E-01	−0.49	−0.48	−0.53	−0.5	−0.48	−0.47	−0.47	−0.47	−0.47
4.20E-01	−0.49	−0.49	−0.53	−0.5	−0.48	−0.47	−0.47	−0.47	−0.47
4.40E-01	−0.49	−0.49	−0.53	−0.5	−0.48	−0.47	−0.47	−0.47	−0.47
4.60E-01	−0.49	−0.49	−0.53	−0.5	−0.48	−0.47	−0.47	−0.47	−0.47
4.80E-01	−0.49	−0.49	−0.53	−0.5	−0.48	−0.47	−0.47	−0.47	−0.47
5.00E-01	−0.49	−0.49	−0.53	−0.5	−0.48	−0.47	−0.47	−0.47	−0.47
5.20E-01	−0.49	−0.49	−0.53	−0.5	−0.48	−0.47	−0.47	−0.47	−0.47
5.40E-01	−0.49	−0.49	−0.53	−0.5	−0.48	−0.47	−0.47	−0.47	−0.47
5.60E-01	−0.49	−0.49	−0.53	−0.5	−0.48	−0.47	−0.47	−0.47	−0.47
5.80E-01	−0.49	−0.49	−0.53	−0.5	−0.48	−0.47	−0.47	−0.47	−0.47
6.00E-01	−0.49	−0.49	−0.53	−0.5	−0.48	−0.47	−0.47	−0.47	−0.47
6.20E-01	−0.49	−0.49	−0.53	−0.5	−0.48	−0.47	−0.47	−0.47	−0.47
6.40E-01	−0.49	−0.49	−0.53	−0.5	−0.48	−0.47	−0.47	−0.47	−0.47
6.60E-01	−0.49	−0.49	−0.53	−0.5	−0.48	−0.47	−0.47	−0.47	−0.47
6.80E-01	−0.49	−0.49	−0.53	−0.5	−0.48	−0.47	−0.47	−0.47	−0.47
7.00E-01	−0.49	−0.49	−0.53	−0.5	−0.48	−0.47	−0.47	−0.47	−0.47
7.20E-01	−0.49	−0.49	−0.53	−0.5	−0.48	−0.47	−0.47	−0.47	−0.47
7.40E-01	−0.49	−0.49	−0.53	−0.5	−0.48	−0.47	−0.47	−0.47	−0.47
7.60E-01	−0.49	−0.49	−0.53	−0.5	−0.48	−0.47	−0.47	−0.47	−0.47
7.80E-01	−0.49	−0.49	−0.53	−0.5	−0.48	−0.47	−0.47	−0.47	−0.47
8.00E-01	−0.49	−0.49	−0.53	−0.5	−0.48	−0.47	−0.47	−0.47	−0.47
8.20E-01	−0.49	−0.49	−0.53	−0.5	−0.48	−0.47	−0.47	−0.47	−0.47
8.40E-01	−0.49	−0.49	−0.53	−0.5	−0.48	−0.47	−0.47	−0.47	−0.47
8.60E-01	−0.49	−0.49	−0.53	−0.5	−0.48	−0.47	−0.47	−0.47	−0.47
8.80E-01	−0.49	−0.49	−0.53	−0.5	−0.48	−0.47	−0.47	−0.47	−0.47
9.00E-01	−0.49	−0.49	−0.53	−0.5	−0.48	−0.47	−0.47	−0.47	−0.47
9.20E-01	−0.49	−0.49	−0.53	−0.5	−0.48	−0.47	−0.47	−0.47	−0.47
9.40E-01	−0.49	−0.49	−0.53	−0.5	−0.48	−0.47	−0.47	−0.47	−0.47
9.60E-01	−0.49	−0.49	−0.53	−0.5	−0.48	−0.47	−0.47	−0.47	−0.47
9.80E-01	−0.49	−0.49	−0.53	−0.5	−0.48	−0.47	−0.47	−0.47	−0.47
1.00E+00	−0.49	−0.49	−0.53	−0.5	−0.48	−0.47	−0.47	−0.47	−0.47
1.02E+00	−0.49	−0.49	−0.53	−0.5	−0.48	−0.47	−0.47	−0.47	−0.47
1.04E+00	−0.49	−0.49	−0.53	−0.5	−0.48	−0.47	−0.47	−0.47	−0.47
1.06E+00	−0.49	−0.49	−0.53	−0.5	−0.48	−0.47	−0.47	−0.47	−0.47
1.08E+00	−0.49	−0.49	−0.53	−0.5	−0.48	−0.47	−0.47	−0.47	−0.47
1.10E+00	−0.49	−0.49	−0.53	−0.5	−0.48	−0.47	−0.47	−0.47	−0.47
1.12E+00	−0.49	−0.49	−0.53	−0.5	−0.48	−0.47	−0.47	−0.47	−0.47
1.14E+00	−0.49	−0.49	−0.53	−0.5	−0.48	−0.47	−0.47	−0.47	−0.47
1.16E+00	−0.49	−0.49	−0.53	−0.5	−0.48	−0.47	−0.47	−0.47	−0.47
1.18E+00	−0.49	−0.49	−0.53	−0.5	−0.48	−0.47	−0.47	−0.47	−0.47
1.20E+00	−0.49	−0.49	−0.53	−0.5	−0.48	−0.47	−0.47	−0.47	−0.47
1.22E+00	−0.49	−0.49	−0.53	−0.5	−0.48	−0.47	−0.47	−0.47	−0.47
1.24E+00	−0.49	−0.49	−0.53	−0.5	−0.48	−0.47	−0.47	−0.47	−0.47
1.26E+00	−0.49	−0.49	−0.53	−0.5	−0.48	−0.47	−0.47	−0.47	−0.47
1.28E+00	−0.49	−0.49	−0.53	−0.5	−0.48	−0.47	−0.47	−0.47	−0.47
1.30E+00	−0.49	−0.49	−0.53	−0.5	−0.48	−0.47	−0.47	−0.47	−0.47
1.32E+00	−0.49	−0.49	−0.53	−0.5	−0.48	−0.47	−0.47	−0.47	−0.47
1.34E+00	−0.49	−0.49	−0.53	−0.5	−0.48	−0.47	−0.47	−0.47	−0.47
1.36E+00	−0.49	−0.49	−0.53	−0.5	−0.48	−0.47	−0.47	−0.47	−0.47
1.38E+00	−0.49	−0.49	−0.53	−0.5	−0.48	−0.47	−0.47	−0.47	−0.47
1.40E+00	−0.49	−0.49	−0.53	−0.5	−0.48	−0.47	−0.47	−0.47	−0.47
1.42E+00	−0.49	−0.49	−0.53	−0.5	−0.48	−0.47	−0.47	−0.47	−0.47
1.44E+00	−0.49	−0.49	−0.53	−0.5	−0.48	−0.47	−0.47	−0.47	−0.47
1.46E+00	−0.49	−0.49	−0.53	−0.5	−0.48	−0.47	−0.47	−0.47	−0.47
1.48E+00	−0.49	−0.49	−0.53	−0.5	−0.48	−0.47	−0.47	−0.47	−0.47
1.50E+00	−0.49	−0.49	−0.53	−0.5	−0.48	−0.47	−0.47	−0.47	−0.47
1.52E+00	−0.49	−0.49	−0.53	−0.5	−0.48	−0.47	−0.47	−0.47	−0.47
1.54E+00	−0.49	−0.49	−0.53	−0.5	−0.48	−0.47	−0.47	−0.47	−0.47
1.56E+00	−0.49	−0.49	−0.53	−0.5	−0.48	−0.47	−0.47	−0.47	−0.47
1.58E+00	−0.49	−0.49	−0.53	−0.5	−0.48	−0.47	−0.47	−0.47	−0.47
1.60E+00	−0.49	−0.49	−0.53	−0.5	−0.48	−0.47	−0.47	−0.47	−0.47
1.62E+00	−0.49	−0.49	−0.53	−0.5	−0.48	−0.47	−0.47	−0.47	−0.47
1.64E+00	−0.49	−0.49	−0.53	−0.5	−0.48	−0.47	−0.47	−0.47	−0.47
1.66E+00	−0.49	−0.49	−0.53	−0.5	−0.48	−0.47	−0.47	−0.47	−0.47
1.68E+00	−0.49	−0.49	−0.53	−0.5	−0.48	−0.47	−0.47	−0.47	−0.47
1.70E+00	−0.49	−0.49	−0.53	−0.5	−0.48	−0.47	−0.47	−0.47	−0.47
1.72E+00	−0.49	−0.49	−0.53	−0.5	−0.48	−0.47	−0.47	−0.47	−0.47
1.74E+00	−0.49	−0.49	−0.53	−0.5	−0.48	−0.47	−0.47	−0.47	−0.47
1.76E+00	−0.49	−0.49	−0.53	−0.5	−0.48	−0.47	−0.47	−0.47	−0.47
1.78E+00	−0.49	−0.49	−0.53	−0.5	−0.48	−0.47	−0.47	−0.47	−0.47
1.80E+00	−0.49	−0.49	−0.53	−0.5	−0.48	−0.47	−0.47	−0.47	−0.47
1.82E+00	−0.49	−0.49	−0.53	−0.5	−0.48	−0.47	−0.47	−0.47	−0.47
1.84E+00	−0.49	−0.49	−0.53	−0.5	−0.48	−0.47	−0.47	−0.47	−0.47
1.86E+00	−0.49	−0.49	−0.53	−0.5	−0.48	−0.47	−0.47	−0.47	−0.47
1.88E+00	−0.49	−0.49	−0.53	−0.5	−0.48	−0.47	−0.47	−0.47	−0.47
1.90E+00	−0.49	−0.49	−0.53	−0.5	−0.48	−0.47	−0.47	−0.47	−0.47
1.92E+00	−0.49	−0.49	−0.53	−0.5	−0.48	−0.47	−0.47	−0.47	−0.47
1.94E+00	−0.49	−0.49	−0.53	−0.5	−0.48	−0.47	−0.47	−0.47	−0.47
1.96E+00	−0.49	−0.49	−0.53	−0.5	−0.48	−0.47	−0.47	−0.47	−0.47
1.98E+00	−0.49	−0.49	−0.53	−0.5	−0.48	−0.47	−0.47	−0.47	−0.47
2.00E+00	−0.49	−0.49	−0.53	−0.5	−0.48	−0.47	−0.47	−0.47	−0.47
2.02E+00	−0.49	−0.49	−0.53	−0.5	−0.48	−0.47	−0.47	−0.47	−0.47
2.04E+00	−0.49	−0.49	−0.53	−0.5	−0.48	−0.47	−0.47	−0.47	−0.47
2.06E+00	−0.49	−0.49	−0.53	−0.5	−0.48	−0.47	−0.47	−0.47	−0.47
2.08E+00	−0.49	−0.49	−0.53	−0.5	−0.48	−0.47	−0.47	−0.47	−0.47
2.10E+00	−0.49	−0.49	−0.53	−0.5	−0.48	−0.47	−0.47	−0.47	−0.47
2.12E+00	−0.49	−0.49	−0.53	−0.5	−0.48	−0.47	−0.47	−0.47	−0.47
2.14E+00	−0.49	−0.49	−0.53	−0.5	−0.48	−0.47	−0.47	−0.47	−0.47
2.16E+00	−0.49	−0.49	−0.53	−0.5	−0.48	−0.47	−0.47	−0.47	−0.47
2.18E+00	−0.49	−0.49	−0.53	−0.5	−0.48	−0.47	−0.47	−0.47	−0.47
2.20E+00	−0.49	−0.49	−0.53	−0.5	−0.48	−0.47	−0.47	−0.47	−0.47
2.22E+00	−0.49	−0.49	−0.53	−0.5	−0.48	−0.47	−0.47	−0.47	−0.47
2.24E+00	−0.49	−0.49	−0.53	−0.5	−0.48	−0.47	−0.47	−0.47	−0.47
2.26E+00	−0.49	−0.49	−0.53	−0.5	−0.48	−0.47	−0.47	−0.47	−0.47
2.28E+00	−0.49	−0.49	−0.53	−0.5	−0.48	−0.47	−0.47	−0.47	−0.47
2.30E+00	−0.49	−0.49	−0.53	−0.5	−0.48	−0.47	−0.47	−0.47	−0.47
2.32E+00	−0.49	−0.49	−0.53	−0.5	−0.48	−0.47	−0.47	−0.47	−0.47
2.34E+00	−0.49	−0.49	−0.53	−0.5	−0.48	−0.47	−0.47	−0.47	−0.47
2.36E+00	−0.49	−0.49	−0.53	−0.5	−0.48	−0.47	−0.47	−0.47	−0.47
2.38E+00	−0.49	−0.49	−0.53	−0.5	−0.48	−0.47	−0.47	−0.47	−0.47
2.40E+00	−0.49	−0.49	−0.53	−0.5	−0.48	−0.47	−0.47	−0.47	−0.47
2.42E+00	−0.49	−0.49	−0.53	−0.5	−0.48	−0.47	−0.47	−0.47	−0.47
2.44E+00	−0.49	−0.49	−0.53	−0.5	−0.48	−0.47	−0.47	−0.47	−0.47
2.46E+00	−0.49	−0.49	−0.53	−0.5	−0.48	−0.47	−0.47	−0.47	−0.47
2.48E+00	−0.49	−0.49	−0.53	−0.5	−0.48	−0.47	−0.47	−0.47	−0.47
2.50E+00	−0.49	−0.49	−0.53	−0.5	−0.48	−0.47	−0.47	−0.47	−0.47
2.52E+00	−0.49	−0.49	−0.53	−0.5	−0.48	−0.47	−0.47	−0.47	−0.47
2.54E+00	−0.49	−0.49	−0.53	−0.5	−0.48	−0.47	−0.47	−0.47	−0.47
2.56E+00	−0.49	−0.49	−0.53	−0.5	−0.48	−0.47	−0.47	−0.47	−0.47
2.58E+00	−0.49	−0.49	−0.53	−0.5	−0.48	−0.47	−0.47	−0.47	−0.47
2.60E+00	−0.49	−0.49	−0.53	−0.5	−0.48	−0.47	−0.47	−0.47	−0.47
2.62E+00	−0.49	−0.49	−0.53	−0.5	−0.48	−0.47	−0.47	−0.47	−0.47
2.64E+00	−0.49	−0.49	−0.53	−0.5	−0.48	−0.47	−0.47	−0.47	−0.47
2.66E+00	−0.49	−0.49	−0.53	−0.5	−0.48	−0.47	−0.47	−0.47	−0.47
2.68E+00	−0.49	−0.49	−0.53	−0.5	−0.48	−0.47	−0.47	−0.47	−0.47
2.70E+00	−0.49	−0.49	−0.53	−0.5	−0.48	−0.47	−0.47	−0.47	−0.47
2.72E+00	−0.49	−0.49	−0.53	−0.5	−0.48	−0.47	−0.47	−0.47	−0.47
2.74E+00	−0.49	−0.49	−0.53	−0.5	−0.48	−0.47	−0.47	−0.47	−0.47
2.76E+00	−0.49	−0.49	−0.53	−0.5	−0.48	−0.47	−0.47	−0.47	−0.47
2.78E+00	−0.49	−0.49	−0.53	−0.5	−0.48	−0.47	−0.47	−0.47	−0.47
2.80E+00	−0.49	−0.49	−0.53	−0.5	−0.48	−0.47	−0.47	−0.47	−0.47
2.82E+00	−0.49	−0.49	−0.53	−0.5	−0.48	−0.47	−0.47	−0.47	−0.47
2.84E+00	−0.49	−0.49	−0.53	−0.5	−0.48	−0.47	−0.47	−0.47	−0.47
2.86E+00	−0.49	−0.49	−0.53	−0.5	−0.48	−0.47	−0.47	−0.47	−0.47
2.88E+00	−0.49	−0.49	−0.53	−0.5	−0.48	−0.47	−0.47	−0.47	−0.47
2.90E+00	−0.49	−0.49	−0.53	−0.5	−0.48	−0.47	−0.47	−0.47	−0.47
2.92E+00	−0.49	−0.49	−0.53	−0.5	−0.48	−0.47	−0.47	−0.47	−0.47
2.94E+00	−0.49	−0.49	−0.53	−0.5	−0.48	−0.47	−0.47	−0.47	−0.47
2.96E+00	−0.49	−0.49	−0.53	−0.5	−0.48	−0.47	−0.47	−0.47	−0.47
2.98E+00	−0.49	−0.49	−0.53	−0.5	−0.48	−0.47	−0.47	−0.47	−0.47

## 2 Experimental Design, Materials and Methods

Bi- nano additives made up of crushed coconut shell with egg shell were used as materials [1,2,3,4,5]. The egg shells having a number of 483 were crumpled into smaller pieces using a mortar and a pestle were amassed and put into a crucible blast furnace which has the thermal heat capacity of up to 2300 °C, but for this experiment, the furnace was set to burn for 3 h at a temperature of 2000 °C. The ashes were allowed to cool for more than 10 h and was properly sieved using a sieve shaker to separate outsized particles from the desired sized particles. 50 g of the ashes was weighed on the mass balance before put into a beaker filled with 100 g of salt and 1000 ml of distilled water which was measured with a measuring cylinder. This composition was thoroughly mixed using a magnetic stirrer in the beaker and placing the sample on a Stuart hot plate. This process was repeated twelve times for the same sample and collected in plastic transparent kegs. The solution was left to settle in the keg for a period time of 24 h before centrifugation process took place. The drying and calcination process involved putting the powder inside the oven at 500 °C for two days.

The coconut shells (i.e. the brown part of the coconut fruit) were sourced from a local coconut drink seller in the market. The shells were then sorted and the useful parted under the sun for 3 days. It was ground into rough coarse powder using a hardened steel crusher and a disc grinder and it was then further reduced to fine powder using a planetary ball mill. After the Coconut shell powder was produced, it was burnt in a Muffle furnace at a temperature of 900 °C for about 2 h which produced coconut shell ashes, a raw material for the synthesis process. The hot coconut shell ash was then left to cool for about 12 h. The synthesis process consisted of three stages which are reaction, centrifugation and calcination processes. The nano particles gotten from both egg and coconut shell were admixed together at proportional rates (shown in Table 1) before been mixed with distilled water at gram per litre.

10 mild steel plate samples of dimensions 20 mm x 20 mm were dry abraded with emery papers of 80, 180, 320 and 600 μm and thereafter immersed in 100 ml of the nano-fluid at different concentrations. The coconut shell and egg shell ratio used was 90-10, 80-20, 70-30, 60-40, 50-50, 40-60, 30-70, 20-80 and 10-90 respectively. For this data, initial and final weights of mild steel coupons were taken during immersion test and the corrosion rate calculated in mm/year via Eq. 1 [6,7].

(1) $$\text{Corrosion}\;\text{Rate}=87.6\times \left(\frac{W}{DAT}\right)$$

Where W is weight loss in mg, D is metal density in g/cm^3^, A is sample area in cm^2^, T is exposure time in hours. A Digi-Ivy Potentiostat was used for open circuit potential measurements. Duplicate tests were carried out in order to determine the reproducibility of the data.

## Declaration of Competing Interest

None.

## References

[1] Udo M.O., Esezobor D.E., Apeh F.I., Afolalu S.A. (2018). Factors affecting ballability of mixture iron ore concentrates and iron oxide bearing wastes in metallurgical processing. J. Ecol. Eng. Vol.

[2] Oyinbo S.T., Ikumapayi O.M., Ajiboye J.S., Afolalu S.A. (2015). Numerical simulation of axisymmetric and asymmetric extrusion process using finite element method. Int. J. Sci. Eng. Res..

[3] Afolalu S.A., Abioye A.A., Udo M.O., Adetunji O.R., Ikumapayi O.M., Adejuyigbe S.B. (2018). Data showing the effects of temperature and time variances on nano-additives treatment of mild steel during machining. Data in Brief.

[4] Adetunji O.R., Musa A.A., Afolalu S.A. (2015). Computational Modelling of chromium steel in high temperature applications. Int. J. Innov. Appl. Stud..

[5] Adetunji O.R., Ude O.O., Kuye S.I, Dare E.O., Alamu K.O., Afolalu S.A. (2016). Potentiodynamic polarization of brass, stainless and coated mild steel in 1m sodium chloride solution. Int. J. Eng. Res. Africa.

[6] ASTM G1-03 (2003). Standard Practice for Preparing, Cleaning and Evaluating Corrosion Test Specimens, Annual Book of ASTM Standards.

[7] Joseph O.O., Ajayi J.A. (2020). Data on chloride-related electrochemical deterioration of micro-alloyed steel in E20 simulated fuel ethanol blend. Chem. Data Collect..

